# Uptake and effectiveness of influenza vaccine in those aged 65 years and older in the United Kingdom, influenza seasons 2010/11 to 2016/17

**DOI:** 10.2807/1560-7917.ES.2018.23.39.1800092

**Published:** 2018-09-27

**Authors:** Richard G Pebody, Fiona Warburton, Nick Andrews, Mary Sinnathamby, Ivelina Yonova, Arlene Reynolds, Chris Robertson, Simon Cottrell, Muhammad Sartaj, Rory Gunson, Matthew Donati, Catherine Moore, Joanna Ellis, Simon de Lusignan, Jim McMenamin, Maria Zambon

**Affiliations:** 1 Public Health England, London, United Kingdom; 2 University of Surrey, Guildford, United Kingdom; 3 Royal College of General Practitioners, London, United Kingdom; 4 Health Protection Scotland, Glasgow, United Kingdom; 5 University of Strathclyde, Glasgow, United Kingdom; 6 Public Health Wales, Cardiff, United Kingdom; 7 Public Health Agency Northern Ireland, Belfast, United Kingdom; 8 West of Scotland Specialist Virology Centre, Glasgow, United Kingdom; 9 Public Health England, Bristol, United Kingdom

**Keywords:** United Kingdom, viral infections, influenza, vaccine uptake and effectiveness, immunisation

## Abstract

In 2016/17, seasonal influenza vaccine was less effective in those aged 65 years and older in the United Kingdom. We describe the uptake, influenza-associated mortality and adjusted vaccine effectiveness (aVE) in this age group over influenza seasons 2010/11–2016/17. **Methods:** Vaccine uptake in 2016/17 and five previous seasons were measured using a sentinel general practitioners cohort in England; the test-negative case-control design was used to estimate pooled aVE by subtype and age group against laboratory-confirmed influenza in primary care from 2010–2017. **Results:** Vaccine uptake was 64% in 65–69-year-olds, 74% in 70–74-year-olds and 80% in those aged 75 and older. Overall aVE was 32.5% (95% CI: 11.6 to 48.5); aVE by sub-type was 60.8% (95% CI: 33.9 to 76.7) and 50.0% (95% CI: 21.6 to 68.1) against influenza A(H1N1)pdm09 and influenza B, respectively, but only 5.6% (95% CI: - 39.2 to 35.9) against A(H3N2). Against all laboratory-confirmed influenza aVE was 45.2% (95% CI: 25.1 to 60.0) in 65–74 year olds; - 26.2% (95% CI: - 149.3 to 36.0) in 75–84 year olds and - 3.2% (95% CI: - 237.8 to 68.5) in those aged 85 years and older. Influenza-attributable mortality was highest in seasons dominated by A(H3N2). **Conclusions:** Vaccine uptake with non-adjuvanted, normal-dose vaccines remained high, with evidence of effectiveness against influenza A(H1N1)pdm09 and B, though poor against A(H3N2), particularly in those aged 75 years and older. Forthcoming availability of newly licensed vaccines with wider use of antivirals can potentially further improve prevention and control of influenza in this group.

## Introduction

The United Kingdom (UK), like many other countries in Europe, North America and Australasia, has a longstanding inactivated influenza vaccine programme including for all those over 65 years of age. The UK universal influenza vaccine programme for those aged 65 years and older was first started in 2000/01 [1], following several seasons of intense A(H3N2) activity associated with substantial morbidity and excess mortality particularly in this older age group [2]. This together with vaccination effectiveness (VE) estimates at that time [3] informed the decision to offer influenza vaccine free of charge to all individuals aged 65 years and older in addition to high-risk groups, such as those with underlying chronic respiratory and cardiovascular disease.

Following this change in vaccine policy, influenza vaccine uptake for those aged 65 years and older has increased, reaching a high point in excess of the World Health Organization (WHO) and European Council recommendation of 75% in 2005/06 in England. Although there has been a gradual decline in uptake since that time, coverage was still 70.5% in 2016/17, ranging from 66.6% to 74.5% in other UK countries [4]. Although excess influenza-associated mortality has reduced since the high levels observed during the 1990s, there is still a substantial disease burden on the population, particularly in those aged 65 years and older and most often during seasons with intense influenza A(H3N2) activity [5]. A recently published UK VE study for the 2016/17 season, when influenza A(H3N2) was again the dominant circulating strain, found moderate to good VE in children and younger adults but no evidence of effectiveness in those aged 65 years and older [6]. Newer vaccines that provide direct protection against influenza e.g. adjuvanted, higher dose, cell-based or are recombinant [7] are becoming increasingly available. These, in combination with indirect protection from the progressive rollout of the UK childhood influenza vaccine programme, means that the potential role of these alternative interventions in reducing disease burden in this age group needs to be further explored [7].

The aim of this study was to further describe the recent uptake of influenza vaccine in those aged 65 years and older (including prior vaccine history) to measure influenza-associated mortality in this age group and to estimate the effectiveness of influenza vaccine over the period 2010–2017. The results will help inform optimal approaches to further mitigate the impact of influenza in this enlarging group.

## Methods

### Influenza vaccine uptake in those aged 65 years and older, influenza season 2016/17

The previously described Royal College of General Practitioners (RCGP) Research Surveillance Centre (RSC) VE cohort was used to measure vaccine uptake and obtain prior vaccine history [8]. The RCGP RSC cohort is composed of the entire registered population in participating RSC sentinel practices across England. The entry date to the cohort was 1 September 2016 and exit date was 31 May 2017, with influenza vaccination history for the five previous seasons. The cohort was restricted to patients that were alive, aged 65–100 years of age and registered for at least 1 year with any of the practices during the study period.

### Influenza-associated mortality in those aged 65 years and older, influenza seasons 2011/12–2016/17

Using the weekly number of all-cause death registrations for those aged 65 years and older in England, available from the Office for National Statistics (ONS) for the period September 2011–September 2017, a previously described multiplicative Poisson regression model (the FluMoMo model) was used to estimate the number of deaths associated with influenza [9]. In brief, the model baseline adjusts for seasonal and temporal variation by including a sine curve and trend. In addition, the model simultaneously controls for influenza activity (IA) and extreme temperature (ET). As the impact of influenza may vary by season, IA is included separately from each influenza season and calculated as weekly influenza-like illness (ILI) consultation rate multiplied by the swab positivity rate. While the effect of influenza is allowed to vary each season, the effect of extreme temperature is assumed constant over time, and is included in the model as two variables; a summer effect (s) and a winter effect (w), both defined as departures from normal weekly average temperatures.

E[log(X_t_)] = β_0_ + β_1_*trend(t) + β_2_*seasonality(t) + Σβ_is_*IA_t,is_ + β_s_*ET_s,t_+ β_w_*ET_w,t_

where β_0_ + β_1_*trend(t) + β_2_*seasonality(t) comprise the baseline, and the remainder impacts of IA and ET.

The FluMoMo model was run separately for each age group (65–74 years and those aged 75 years and older) and by sub-type, (influenza A and B).

### Vaccine effectiveness in those aged 65 years and older, influenza seasons 2010/11–2016/17

#### Data sources and study population

The Test Negative Case (TNCC) control design has been used to measure vaccine effectiveness against laboratory-confirmed influenza infection that has resulted in acute influenza-like illness consultation in primary care. Five sentinel primary care surveillance schemes across the UK provided swabbing data (two from England, one each from Northern Ireland, Scotland and Wales). Details of these swabbing schemes have been published previously [10]. The study period was from 1 October 2010 until 19 March 2017: a period when non-adjuvanted, non-high dose inactivated influenza vaccine (IIV) was administered to those aged 65 years and older through primary care in the UK. Cases were defined as people aged 65 years and older consulting their general practitioner (GP) with an acute ILI who were swabbed and tested positive for influenza A or B with real-time polymerase chain reaction (RT-PCR). Controls were people aged 65 years and older with ILI who tested negative for influenza A and B. ILI was defined as an acute respiratory illness with physician-diagnosed fever or history of fever in the previous 7 days. Patients were swabbed as part of clinical care with verbal consent. A standardised questionnaire was completed by the patient’s GP at the time of swabbing collecting key demographic, clinical and epidemiological information including date of birth, date of onset of illness, date of the swab, date of vaccination that season, sex and underlying clinical risk group as per the Green Book (national immunisation) guidance [11].

Laboratory confirmation was undertaken using RT-PCR assays for circulating influenza A, influenza B and other respiratory viruses at the national laboratory [12,13]. Samples in England were sent to the Public Health Agency England (PHE) Reference Virus Unit, Colindale (RCGP RSC scheme) or one of the specialist regional microbiology laboratories (SMN scheme). Samples in Wales were sent to the Public Health Wales Specialist Virology Centre, Cardiff and samples in Scotland were sent to the West of Scotland Specialist Virology Centre, Glasgow (HPS scheme). In Northern Ireland samples were sent to the Regional Virus Laboratory, Belfast.

#### Vaccination status

Cases and controls were defined as vaccinated if the date of vaccination that season was 14 or more days before onset of ILI. Cases and controls with less than 14 days between vaccination and onset of illness were excluded, as their immune status was unclear. If the date of vaccination was missing it was assigned 15 October, which was the median of all known vaccination dates. If the date of onset of symptoms was missing, then the individual was excluded. Respiratory samples with more than 7 days delay between onset of illness and sample collection were excluded, as the sensitivity of the PCR test decreases over longer intervals between onset and sampling.

### Statistical analysis

Average VE over the entire seven-season period (2010/11–2016/17) was estimated as 1-(OR) using multivariable logistic regression models with influenza A(H3N2), A(H1N1)pdm09 or influenza B PCR results as outcomes and seasonal vaccination status as the linear predictor. Age (coded into three age groups 65–74, 75–84 and  85 years and older), sex, season, clinical risk group, surveillance scheme (RCGP, SMN, HPS, Public Health Wales, Northern Ireland) and date of sample collection (month) were investigated as potential confounding variables.

To investigate whether the VE changed in relation to time since vaccination, influenza VE was stratified by time since vaccination (< 3 months, ≥ 3 months). To investigate whether the VE changed with age, an interaction term between vaccination and age (as a continuous variable) was included in the model. To test the significance of changes in VE with the time since vaccination, multivariable logistic regression was performed with time between vaccination and onset of symptoms (days) included as a continuous variable. As testing for evidence of waning immunity was one of the study objectives, multiple testing adjustments were not made.

All statistical analyses were carried out in Stata version 13 (StataCorp, College Station, Texas).

## Results

### Influenza vaccine uptake in those aged 65 years and older, influenza season 2016/17

During influenza season 2016/17, among 65–69-year-olds in England, influenza vaccine uptake was 64% (55,818/87,566) compared with 74% (52,873/71,298) in 70–74 year olds and 80% (71,072/89,228) for those aged between 75–84 years or aged 85 years and older (28,143/35,097). The majority (99,215/124,325) of people vaccinated in 2016/17 had received one or more doses of IIV in the previous 5 years, with most of those aged over 70 years having received all five doses of IIV that they were eligible for over the previous five seasons (Table 1).

**Table 1:** Influenza vaccine uptake and prior vaccine history by age group, Royal College of General Practitioners participating practices, England, influenza seasons 2010/11–2016/17 (n = 283,188)

Number of prior vaccine doses	65–69 years N = 87,565		70–74 years N = 71,298		75–84 years N = 89,228		≥ 85 years N = 35,097		All age N = 283,189	
	Unvacc	Vacc	Unvacc	Vacc	Unvacc	Vacc	Unvacc	Vacc	Unvacc	Vacc
0	23,403	5,467	11,457	1,482	10,956	1,673	3,694	655	49,510	9,277
1	3,915	6,868	2,145	1,556	1,886	1,531	678	713	8,624	10,668
2	1,831	7,683	1,469	2,733	1,373	2,672	555	1,183	5,228	14,271
3	1,178	8,115	1,144	3,972	1,090	3,551	469	1,538	3,881	17,176
4	820	8,925	1,088	6,564	1,181	6,788	571	2,873	3,660	25,150
5	601	18,759	1,122	36,566	1,670	54,857	987	21,181	4,380	131,363
**All**	**31,748**	**55,817**	**18,425**	**52,873**	**18,156**	**71,072**	**6,954**	**28,143**	**75,283**	**207,905**

Unvacc: unvaccinated; Vacc: vaccinated.

### Influenza-associated mortality in those aged 65 years and older, influenza seasons 2011/12–2016/17

During influenza seasons 2011/12–2016/17 in England, influenza-associated mortality estimates were higher in both the 65–74-year-olds and those aged 75 years and older during influenza A(H3N2) compared with A(H1N1)pdm09 dominated seasons (Table 2). Over five seasons with available data, average annual mortality rates for influenza A and B in those aged 75 years and older were 186.3 and 44.7 per 100,000 population vs 22.5 and 5.0 for 65–74-year-olds, respectively.

**Table 2:** Average annual influenza-associated mortality rates by age groups and influenza type over five seasons, England, influenza seasons 2011/12–2016/17

Influenza season and dominant and co-circulating subtype(s)	A		B	
	Influenza-attributable deaths (95% CI)	Mortality rate per 100,000 population (95% CI)	Influenza-attributable deaths (95% CI)	Mortality rate per 100,000 population (95% CI)
**65–74 years**				
2011/12	368	8.0	428	9.3
A(H3N2)/B	(303 to 438)	(7.0 to 9.5)	(344 to 519)	(7.5 to 11.3)
2012/13	590	12.2	13	0.3
A(H3N2)/B	(500 to 684)	(10.3 to 14.1)	(0 to 45)	(0.0 to 0.9)
2013/14	16	0.3	22	0.4
A(H1N1)pdm09	(0–48)	(0.0 to 1.0)	(3 to 50)	(0.1 to 1.0)
2014/15	2,378	46.1	143	2.8
A(H3N2)/B	(2,153 to 2,611)	(41.7–50.6)	(100 to 191)	(1.9 to 3.7)
2015/16	1,825	34.5	876	16.6
A(H1N1)pmd09/B	(1,701 to 1,952)	(32.2 to 36.9)	(782 to 973)	(14.8 to 18.4)
2016/17	1,828	33.8	20	0.4
A(H3N2)/B	(1,648 to 2,014)	(30.4 to 37.2)	(0 to 71)	(0.0 to 1.3)
**Aged 75 and older years**				
2011/12	3,122	75.5	3,098	74.9
A(H3N2)/B	(2,737 to3,523)	(66.2 to 85.1)	(2,545 to 3,686)	(61.5 to 89.1)
2012/13	6,103	144.9	1,232	29.2
A(H3N2)/B	(5,281 to 6,964)	(125.4 to 165.3)	(838 to 1,672)	(19.9 to 39.7)
2013/14^a^	NA^a^	NA^a^	NA^a^	NA^a^
A(H1N1)pdm09				
2014/15	18,680	427.0	3,626	82.9
A(H3N2)/B	(17,657 to 19,722)	(403.6 to 450.8)	(3,105 to 4,173)	(71.0 to 95.4)
2015/16	3,033	68.5	1,194	27.0
A(H1N1)pdm09/B	(2,636 to 3,449)	(59.6 to 77.9)	(875 to 1,544)	(19.8 to 34.9)
2016/17	9,628	215.4	433	9.7
A(H3N2)/B	(8,878 to 10,399)	(198.6 to 232.7)	(175 to 759)	(3.9 to 17.0)

CI: confidence interval; NA: not available.

^a^The model could not attribute deaths for this period.

### Vaccine effectiveness in those aged 65 years and older, influenza seasons 2010/11–2016/17

**Figure 1:**
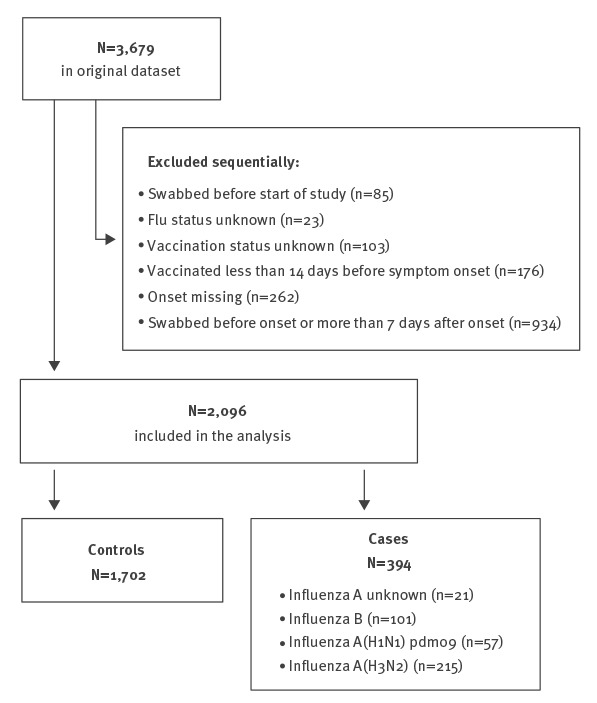
Flowchart to show case and control ascertainment, United Kingdom, influenza seasons 2010/11–2016/17 (n = 2,096)

A total of 3,679 swabs were taken in those over 65 years and older over influenza seasons 2010/11–2016/17, with 2,096 swabs used in the final analysis: of these 1,702 were controls; 101 were flu B, 57 were A(H1N1)pdm09, 215 were A(H3N2) and 21 A non-subtyped. The reasons samples were discarded are outlined in Figure 1. The demographic and clinical details of the cases and controls remaining in the study stratified by swab result are given in Table 3 including by scheme, sex, age, month, onset to swab, year and vaccination status.

**Table 3:** Details for influenza A and B in cases and control aged 65 years and older, test–negative influenza case–control study, United Kingdom, influenza seasons 2010/11–2016/17

Characteristics	Controls	Percent	Influenza type							
			B	Percent	A(H1N1)pdm09	Percent	A(H3N2)	Percent	A (Unknown)	Percent
**Scheme^a^**										
Northern Ireland	64	65.3	4	4.1	3	3.1	20	20.4	7	7.1
RCGP (England)	831	80.9	51	5.0	36	3.5	107	10.4	2	0.2
SMN (England)	122	89.1	3	2.2	3	2.2	9	6.6	0	0.0
Scotland	652	83.3	39	5.0	11	1.4	70	8.9	11	1.4
Wales	33	64.7	4	7.8	4	7.8	9	17.6	1	2.0
**Sex**										
Female	1,040	81.6	53	4.2	30	2.4	137	10.8	14	1.1
Male	655	80.5	48	5.9	27	3.3	77	9.5	7	0.9
Missing	7	87.5	0	0.0	0	0.0	1	12.5	0	0.0
**Age group**										
65–74	1,084	81	76	5.7	42	3.1	124	9.3	13	1.0
75–84	487	87.2	21	3.6	12	2.0	64	10.9	5	0.8
≥ 85	131	78	4	2.4	3	1.8	27	16.1	3	1.8
**Month^a^**										
October	218	98.6	1	0.5	0	0.0	1	0.5	1	0.5
November	283	98.3	3	1.0	1	0.3	1	0.3	0	0.0
December	397	80.4	21	4.3	15	3.0	61	12.3	0	0.0
January	400	77.5	27	5.2	18	3.5	66	12.8	5	1.0
February	223	69.9	21	6.6	11	3.4	58	18.2	6	1.9
March	130	65.0	24	12.0	12	6.0	25	12.5	9	4.5
April	51	87.9	4	6.9	0	0.0	3	5.2	0	0.0
**Onset of symptoms to swab^a^**										
0–1 days	165	77.1	7	3.3	7	3.3	33	15.4	2	0.9
2–4 days	766	78.1	55	5.6	31	3.2	116	11.8	13	1.3
5–7 days	771	85.6	39	4.3	19	2.1	66	7.3	6	0.7
**Influenza season^a^**										
2010/11	222	84.7	24	9.2	16	6.1	0	0.0	0	0.0
2011/12	232	91.7	2	0.8	0	0.0	19	7.5	0	0.0
2012/13	196	73.1	32	11.9	0	0.0	38	14.2	2	0.7
2013/14	144	92.9	0	0.0	0	0.0	3	1.9	8	5.2
2014/15	249	70.9	12	3.4	1	0.3	84	23.9	5	1.4
2015/16	346	84.6	19	4.6	39	9.5	4	1.0	1	0.2
2016/17	313	78.6	12	3.0	1	0.3	67	16.8	5	1.3
**Vaccination^a^**										
Unvaccinated	551	82.4	41	6.1	28	4.2	44	6.6	5	0.7
14–91 days before onset	666	87.7	13	1.7	11	1.4	66	8.7	3	0.4
> 92 days before onset	485	72.6	47	7.0	18	2.7	105	15.7	13	1.9

RCGP: Royal College of General Practitioners Research and Surveillance Centre; SMN: specialist regional microbiology laboratories.

^a^ P value was < 0.001.

When estimating VE, key confounders were adjusted for in a multivariable logistic regression model, all of which (except age and sex) were associated with a positive swab (Table 3). Season and month were identified as confounders for the vaccine effects as the overall estimates changed by more than 5%.

**Figure 2:**
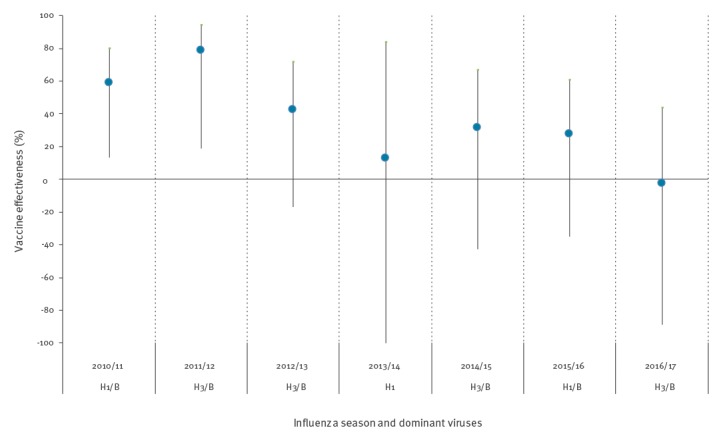
Crude and adjusted vaccine effectiveness estimates in those aged 65 years or older, by influenza season and dominant viruses, United Kingdom, influenza seasons 2010/11–2016/17

The crude and adjusted VE (aVE) estimates in those aged 65 years and older by season are shown in Figure 2, including what was the dominant circulating influenza sub-type that season. The annual aVE point estimate against any laboratory-confirmed influenza infection in those aged 65 years and older ranged from - 2.7% (95% CI: - 88.8 to 44.2) in 2016/17 to 78.8% (95% CI: 18.8 to 94.5) in 2011/12.

Blue dots represent annual adjusted vaccine effectiveness point estimates against any laboratory-confirmed influenza infection in those aged 65 years or older. Black vertical lines are 95% confidence intervals.

Over the seven-season period, for any influenza (A and B), the crude seasonal average VE in those aged 65 years and older was - 12.0% (95% CI: -42.1 to 11.8), which increased to a seasonal average of 32.5% (95% CI: 11.6 to 48.5), when fully adjusted over the entire study period (Table 4). By sub-type aVE in all those aged 65 years and older for A(H3N2) was 5.6% (95% CI: - 39.2 to 35.9), compared with 60.8% (95% CI: 33.9 to 76.7) and 50% (95% CI: 21.6 to 68.1) for A(H1N1)pdm09 and B respectively. By age group, the aVE was 54.2% (95% CI: 25.1 to 60.0) in 65–74-year-olds compared with only -26.3% (95% CI: -149.3 to 36.0) for 75–84-year-olds and -3.2% (95% CI: -237.8 to 68.5) in those aged 85 years and older. The vaccine demonstrated significant effectiveness against A(H1N1)pdm09 and B in 65–74-year-olds, but not in those aged 75 years and older (Table 4).

**Table 4:** Crude and adjusted vaccine effectiveness estimates for influenza by age group and subtype, test–negative case–control study, in those aged 65 years and older, United Kingdom, influenza seasons 2010/11–2016/17

Type/Subtype	Age group	Cases		Controls		Crude VE (95% CI)	Adjusted^a^ VE (95% CI)
		Unvacc	Vacc	Unvacc	Vacc		
**A or B**	All ≥ 65 years	118	276	551	1151	- 12.0 (- 42.1 to 11.8)	32.5 (11.6 to 48.5)
	65–74 years	87	157	370	714	17.0 (- 10.0 to 37.4)	45.2 (25.1 to 60)
	75–84 years	14	88	134	353	- 138.6 (- 333.9 to -31.2)	- 26.3 (- 149.3 to 36.0)
	≥ 85 years	6	31	47	84	- 189.1 (- 643.2 to - 12.4)	- 3.2 (- 237.8 to 68.5)
**A(H1)pdm09**	All ≥ 65 years	33	38	551	1151	44.9 (11.2 to 65.8)	60.8 (33.9 to 76.7)
	65–74 years	28	25	370	714	53.7 (19.5 to 73.4)	68.4 (41.7 to 82.9)
	75–84 years	2	11	134	353	- 108.8 (- 854.3 to 54.3)	- 40.8 (- 643.7 to 73.3)
	≥ 85 years	3	2	47	84	NA	NA
**A(H3N2)**	All ≥ 65 years	47	173	551	1151	- 76.2 (- 147.1 to - 25.7)	5.6 (- 39.2 to 35.9)
	65–74 years	37	91	370	714	- 27.5 (- 90.6 to 14.8)	23.3 (- 20.9 to 51.4)
	75–84 years	7	58	134	353	- 214.5 (- 606.4 to - 40.1)	- 40.9 (- 264.2 to 45.5)
	≥ 85 years	3	24	47	84	- 347.6 (- 1465.8 to - 28.0)	- 91.0 (- 806.6 to 59.8)
**B**	All ≥ 65 years	41	60	551	1151	30.0 (- 5.6 to 53.5)	50.0 (21.6 to 68.1)
	65–74 years	36	40	370	714	42.4 (8.1 to 63.9)	57.9 (29.8 to 74.7)
	75–84 years	5	16	134	353	- 21.5 (- 238.1 to 56.4)	33.3 (- 110.9 to 78.9)
	≥ 85 years	0	4	47	84	NA	NA

CI: confidence interval; NA: Not available; Unvacc: unvaccinated; Vacc: vaccinated.

^a^Adjusted for age group, sex, season, month and surveillance scheme.

Numbers too small to estimate are indicated by NA.

We found that the aVE for all influenza types in those aged 65 years and older reduced by 6.1% for each year older (95% CI: - 10.5 to - 1.9: p = 0.004). For A(H3N2) the aVE reduced by 6.6% (95% CI: - 12.8 to - 0.7: p = 0.03); for A(H1N1)pdm09, by 1.9% (95% CI: - 10.4 to 5.9: p = 0.64) and for influenza B, by 5.5% (95% CI: - 14.1 to 2.5: p = 0.19) for each year older.

For all laboratory-confirmed influenza cases the aVE by time since vaccination, within 3 months of symptom onset, was 44.7% (95% CI: 21.2 to 61.2) compared with 14.5% (95% CI: - 18.2 to 38.2) for more than 3 months between onset and vaccination. For A(H3N2), the aVE was 13.6% (95% CI: - 41.4 to 47.2) if onset was within 3 months of vaccination compared with - 7.1% (95% CI: - 69.7 to 32.5) for more than 3 months since vaccination. For A(H1N1)pdm09, aVE was 66.7% (95% CI: 30.2 to 84.1) compared with 59.1% (95% CI: 23.2 to 78.2) and finally for influenza B, aVE was 71.5% (95% CI: 43.0 to 85.8) compared to 19.1% (95% CI: - 40.2 to 53.3).

## Discussion

We found that despite achieving very high influenza vaccine uptake in those aged 65 years and older, the average annual age group-specific influenza-associated mortality risk in England was highest in those aged 75 years and older over a six-season period and during seasons dominated by circulation of influenza A(H3N2). Over the seven seasons (2010/11–2016/17), the pooled aVE against all-laboratory confirmed influenza diagnosed in primary care across the UK was moderate in those 65 years and older. There was, however, no evidence of statistically significant VE in those aged 75 years and older nor specifically against A(H3N2).

We found evidence of high vaccine uptake, with increasing uptake with age in excess of 80% in those aged 75 years and older, which is comparable to other European countries [14]. England has one of the highest coverage figures in this target group and those aged 75 years and older had received multiple vaccinations over prior seasons. Despite high vaccination coverage over the study period, the influenza-associated mortality was high, particularly in those aged 75 years and older, with the average influenza-associated mortality risk being seven times higher compared with that seen in 65–74-year-olds. Our findings are consistent with an earlier study by Hardelid et al. [5], who while investigating the period 1999–2010 in England, found that the season with the largest number of deaths associated with influenza was 1999/2000. This was a period of time before the introduction of the universal influenza vaccine programme aimed at those aged 65 years and older when the dominant circulating subtype was influenza A(H3N2). We found that influenza-associated mortality has generally been lower since that period, yet there remains statistically significant excess mortality in those aged 65 years and older, particularly in seasons dominated by intense circulation of influenza A(H3N2). Although influenza A is mainly responsible for influenza-associated mortality among those aged 65 years and older, influenza B also poses a risk; Influenza B associated deaths contributed a large proportion of seasonal mortality particularly in the 2011/12 and 2014/15 seasons. For example, in 2014/15 WHO recommended that the annual influenza vaccine be composed of B/Massachusetts/2/2012-like virus of the B/Yamagata lineage, but the dominant circulating strains were antigenically more closely related to B/Phuket/3073/2013, which is another influenza B/Yamagata lineage virus. Reduced aVE against influenza B was observed in those aged 65 years and older that season, suggesting a mismatch [15]. This association between influenza burden and increasing age emphasise the importance of the ageing process on innate and adaptive immunity to influenza infection.

We demonstrate that over the 7 year study period, there was overall evidence of moderate aVE in those aged 65 years and older, though this obscures some important reductions by season, influenza subtype and age group. Immunosenescence is a key contributory factor with overall aVE decreasing with age. Age-related decline in humoral immune response following vaccination is a well-recognised phenomenon [16,17]. Irrespective of vaccination status, reduced humoral responses may be caused or compounded by a reduction in T-cell responsiveness, which may also impair protection against more severe infection [18]. In addition to aVE decreasing with increasing age, we found that although the vaccine protects against influenza A(H1N1)pdm09 and influenza B, it did not against A(H3N2) [19,20]. This lower aVE against laboratory-confirmed A(H3N2) infection in primary care in those aged 65 years and older was previously found for the 2016/17 Northern hemisphere A(H3N2) dominated season—not only in the UK [6] but also elsewhere in Europe [21] and then subsequently in the 2017 winter season in Australia. All of these locations experienced circulation of influenza A(H3N2) 3C.2a sub-clades during this period [22]. There seem to be several factors explaining these observations. Firstly, reduced VE for H3N2 over several years could have been caused by an antigenic mismatch between the circulating strain and vaccine virus, as was seen in 2014/15 [10]. Secondly, there could be amino acid differences between the egg-adapted vaccine strain compared with the wild-type circulating strains leading to a mismatch in induced immunity, as was reported for A(H3N2) in recent seasons [23-25]. Thirdly, we also demonstrate the potentially important role of waning effectiveness within the season, a finding that has been highlighted previously, and which has a variable effect depending on the timing of the influenza epidemic, which is unpredictable between October and March [26]. Finally, we found that vaccinated individuals aged 65 years and older were more likely to have received multiple prior vaccinations, which is consistent with previous studies and indicates that a major predictor of being vaccinated is receiving vaccine the season before [27]. Taken together, these observations suggest that vaccine-induced immunity, particularly to H3N2, may need to be further optimised in those 65 years and older and suggests the necessity for improved vaccine strain selection and vaccine formulation, an observation that has been recognised by WHO.

What potential interventions do we have to improve vaccine performance and potentially reduce the remaining disease burden in this age group? Improving protection through the induction of higher levels of antibody and/or broader repertoires of antibody responses are ways of improving vaccine-induced immunity. Until recently, only normal-dose, non-adjuvanted influenza vaccines were licensed for use in those 65 years and older in the UK, but newer influenza vaccines are becoming increasingly available. MF59 adjuvanted vaccines provide a future alternative option—with effectiveness data from North America and Europe [28-30], suggesting superiority compared with the current generation of influenza vaccines. These vaccines have recently been licensed in the UK. Higher antigen dose inactivated vaccine, which is licensed in the United States for those aged 65 years and older and for which observational data provide encouraging results, provides an alternative option [31,32]. Cell grown and recombinant influenza vaccines are produced independently of eggs and may have higher antigen content, with the latter showing superiority in those aged 50 years and older of age during a mismatched season (2014/15) compared with existing egg-produced vaccines [33]. Quadrivalent influenza vaccines, that provide coverage against both the influenza B lineages, are also becoming more available, providing a solution to the dilemma of which influenza B strain to include in a trivalent vaccine, and provide an incremental improvement on the breadth of induced immunity. However, the long-term goal must be a universal (or near-universal) vaccine that generates a high level of durable and cross-protective immunity against both seasonal and ideally pandemic viruses. In the meantime, more research is needed to understand the effects of egg-induced mutations, antigen dose, and repeated vaccination on clinical protection.

The UK is also in the process of rolling out a universal childhood influenza vaccine programme, targeting children 2-10 years of age, with the intention of providing both direct protection to vaccinated children but also, by reducing their risk of infection and thus transmission, provide indirect protection to vulnerable at-risk groups including those that are 65 years and older. Modelling work by Hodgson et al has demonstrated the projected impact of a paediatric programme on older age group and predicts that the direct and indirect benefits of vaccinating children against influenza should be realised in future years once programme rollout is achieved [34]. Early impact studies conducted in communities where schoolchildren have received influenza vaccine have provided encouraging results of the indirect effects of vaccinating children of primary school-age [35].

It is important to highlight that prompt use of influenza antivirals in vulnerable populations for treatment and prophylaxis purposes is also important to mitigate the impact of severe influenza; particularly during A(H3N2) dominated seasons,. These medicines are, however, currently underutilised in primary care in the UK [36].

One of the key advantages of the TNCC approach is that the design allows control for key biases e.g. the propensity to consult, but confounders still need to be adjusted for. Pooling vaccine effectiveness estimates over a number of seasons has allowed for the further exploration of influenza sub-type and age-specific effects. However, the available numbers of samples limit the precision of some of the stratified estimates e.g. by age group and the ability to assess waning by type/sub-type. Some of the sub-set analyses should thus be interpreted with caution. Other published work has suggested that the TNCC design should control for a possible confounding effect due to frailty in older subjects [37]. Nevertheless, caution should be taken to not over interpret the mortality-associated findings presented here, as we do not know the mortality rates by vaccination status and thus what proportion of deaths might be preventable by more effective influenza vaccines. Further effectiveness studies could start to address this limitation through investigating more severe end-points, such as the impact on hospital admissions. Although our study examined protection against laboratory-confirmed influenza infection in primary care, recent work has also found that influenza vaccine provided poor protection against influenza A(H3N2) confirmed hospitalisation in those 65 years and older, particularly in those aged over 80 years [38]. Similar studies examining protection against influenza-related mortality end-points are, however, urgently required and are planned through linkage of routine healthcare data [39].

## Conclusion

Despite high vaccine uptake with non-adjuvanted, normal-dose inactivated vaccine in older people in the UK, influenza remains an important cause of influenza-associated mortality, particularly due to influenza A(H3N2) and in those aged 75 years and older. The forthcoming policy of administering adjuvanted vaccines to older people in the UK will provide the opportunity to improve the prevention and control of influenza in these older age groups, with guidance for their use particularly in those aged 65 years and older recently published [11]. Further studies are required to understand the performance of these vaccines in comparison to traditional influenza vaccines against a range of end-points.
